# Genome‐Wide Association Studies for 24 Hematological Traits in Production Pigs Before and After Weaning

**DOI:** 10.1002/age.70091

**Published:** 2026-03-26

**Authors:** Jiahong Sun, Emil Ibragimov, Malene Kjelin Morsing, Martin Peter Rydal, Lise Nikolic Nielsen, Zexi Cai, Jens Peter Nielsen, Merete Fredholm, Peter Karlskov‐Mortensen

**Affiliations:** ^1^ Department of Veterinary and Animal Sciences, Faculty of Health and Medical Sciences University of Copenhagen Frederiksberg C Denmark; ^2^ Department of Veterinary Clinical Sciences, Faculty of Health and Medical Sciences University of Copenhagen Frederiksberg C Denmark; ^3^ Center for Quantitative Genetics and Genome Research University of Aarhus Aarhus Denmark

**Keywords:** (landrace × Yorkshire) × duroc crossbred pigs, complete blood count, GWAS, imputation

## Abstract

Hematological traits are essential indicators of an animal's immune status and overall health, reflecting both physiological and pathological conditions. The complete blood count (CBC), a commonly used clinical test, evaluates the concentrations, proportions, and characteristics of various blood cell parameters, providing insights into an animal's current health status. CBC phenotypes are dynamic, influenced not only by health status but also by factors such as physiology, nutrition, environmental conditions, age, and genetic makeup. Previous studies have estimated heritability for specific blood parameters and genome‐wide association studies have identified loci associated with CBC phenotypes. While some loci seem to have similar effects across age and breeds and even across species, other loci seem to have a more breed and/or age‐specific effect. This study extends previous research by conducting genome‐wide association studies (GWAS) of 24 hematological traits measured in the same pig cohort at two ages (25 and 46 days). The GWAS included 1884 pigs at 25 days and 1615 pigs at 46 days, with 1499 pigs sampled at both time points. This analysis identified 57 QTLs for complete blood count (CBC) traits, including 11 lead SNPs associated with more than one trait. All animals were (Landrace × Yorkshire) × Duroc crossbred pigs. Thirty QTLs overlapped with previously reported CBC QTLs in different pig breeds, providing further evidence for shared regulatory regions for CBC traits across pig breeds. Genes in 29 QTLs were associated with comparable CBC phenotypes in humans.

## 1 Introduction

Hematological traits are vital indicators of the immune status and overall health of animals, as they reflect physiological and pathological conditions. A complete blood count (CBC) quantifies major blood cell types—leukocytes, erythrocytes, and platelets—and is widely used to assess immune status, oxygen transport, hemostasis, and bone marrow activity. These parameters therefore serve as practical biomarkers for monitoring health (George‐Gay and Parker 2003).

During the long process of domestication and selection, each pig breed has established distinct production traits, biological features, and breed‐specific genomic variations (Bovo et al. 2020; Wilkinson et al. 2013). However, different pig breeds can still share genetic variants with similar effects on CBC. The PigQTL database reports 2959 SNPs associated with 43 CBC‐related phenotypes across 13 studies (Sun et al. 2025). Beyond animal science, the domestic pig serves as a valuable large‐animal model for studying human diseases due to its remarkable similarity to human physiological traits, including hematological traits (Li et al. 2023). Thus, identifying the genetic underpinnings of CBC traits in pigs will further increase the value of this species as a model for human conditions.

Commercial pig production exposes animals to uniform yet pathogen‐rich environments, making genetic selection for health and robustness increasingly important (Bai et al. 2020). CBC traits are relevant in this context because they can reveal early or subclinical disease processes. Numerous studies in livestock have shown that CBC parameters are associated with production traits and disease. For example, neutrophil granulocyte count significantly increases in dairy cattle and buffalo suffering from chronic diarrhea (Hassan et al. 2022). In calves with diarrhea, hemoglobin, packed cell volume (PCV), and WBCs show significant elevations (Zeena Ebraheem mohammed et al. 2020). In a study looking for correlations between production traits and blood‐based traits, birthweight exhibited the strongest genetic correlations with mean platelet volume (−0.68) and with levels of eosinophils (0.70) (Chinchilla‐Vargas et al. 2020).

Blood cell‐related parameters change over a lifetime. Although there are no comprehensive studies on lifelong hematological changes in pigs, human data provide a useful reference. In humans, newborns have elevated hemoglobin and hematocrit levels due to the relatively low oxygen environment in utero (Ayodele 2016; Driscoll 2015), followed by a postnatal decline (Driscoll 2015). WBC are initially high and dominated by neutrophils (Manroe et al. 1979) but shift to lymphocyte predominance within the first week (Manroe et al. 1979). Platelet counts are within the adult range but the platelets are functionally immature (Driscoll 2015). During infancy, hemoglobin remains low (Driscoll 2015), lymphocytes stay elevated (Ayodele 2016), and iron deficiency becomes a key concern after 6 months (Driscoll 2015).

In pigs, specific developmental stages are known to be physiologically important. The weaning transition—typically around 25 days of age—induces abrupt changes in diet and environment, leading to pronounced physiological stress (Papatsiros et al. 2024). CBC evaluations before and after this stage can therefore indicate resilience and developmental stability. Genetic effects on hematological traits may act throughout life or be restricted to specific stages (Lee 2024).

In this study, we conducted a genome‐wide association analysis (GWAS) of 24 hematological traits in healthy (Landrace × Yorkshire) × Duroc pigs, measured at two time points, that is, the day before weaning (Day 25) and at 46 days of age, that is, 3 weeks after weaning. We also analyzed the ratios between the two measurements to capture dynamic changes over time. Results were compared with findings from pig and human studies to identify shared genetic variants, and we further explored novel loci and potential causative genes through biological and bioinformatics annotation.

## 2 Materials and Methods

### 2.1 Animals

The pigs included in the study were born in 11 batches at a production farm in Denmark in the period March 2023 to July 2024, with 170 sows serving as the biological mothers. No cross‐fostering was performed, ensuring that piglets remained with their biological mothers throughout the lactation period. Piglets were weaned on Day 26. After weaning, piglets from different litters were mixed and housed together in groups of approximately 45 animals until Day 46. The experimental setup and sampling was approved by the Animal Experiments Inspectorate under the Ministry of Food, Agriculture and Fisheries of Denmark for the project “Intestinal health and the robust pig” Journal nr.: 2022‐15‐0201‐01324.

### 2.2 Genotyping and Quality Control

DNA from 2297 pigs was isolated from EDTA‐stabilized blood samples using a salting out procedure (Miller et al. 1988). The GGP Porcine 50 K SNP chip from Neogene was used for genotyping with markers aligned to the reference porcine genome assembly Sus‐scrofa 11.1. The SNP panel comprised 49 456 SNPs in total, of which 46 720 were autosomal. Individuals with high genotype missingness rates (> 5%) and sex misassignment according to the plink ‐‐sex‐check function were removed, resulting in 2177 individuals retained for downstream analyses. Genotype imputation was conducted using the protocol described previously (Cai et al. 2022). Briefly, the genotypes of pigs were phased using Eagle (Loh et al. 2016), and then imputed in two steps: first, from 50 K to high‐density (HD) level by Minimac4 (Fuchsberger et al. 2015), matching the HD level chip Affymetrix Axiom PigHD SNP genotypes (Axiom_PigHDv1, 658 k) of 474 (Landrace × Yorkshire) × Duroc crossbred pigs as the intermediate reference panel. Second, the imputed HD marker set was imputed to whole‐genome sequencing level using 217 WGS individuals of three DanBred commercial pig breeds, 89 Duroc, 61 Landrace, and 67 Yorkshire pigs, using Minimac4 (Fuchsberger et al. 2015). The imputation was performed for autosomal variants only. Before GWAS, genotype quality control was conducted using Plink v1.9 (Purcell et al. 2007). Both original and imputed variants with high genotype missingness rates (> 5%) and variants with minor allele frequency less than 5% were removed. For imputed variants, an additional filter based on imputation accuracy was applied, excluding variants with *R* ^2^ < 0.4 (Figure S1). After quality control, 15 042 117 imputed variants and 44 058 genotyped autosomal variants remained for the analyses.

### 2.3 Blood Collection and Analyses

Samples from a total of 2042 pigs at 25 days of age (D25) and 1754 pigs at 46 days of age (D46) were used for CBC analysis, with 1625 pigs sampled at both time points. Since not all animals included in the CBC analysis retained (after genotype quality control), the final datasets available for genome‐wide association studies (GWAS) comprised 1884 pigs at D25 and 1615 pigs at D46, with 1499 pigs sampled at both time points. None of the pigs received treatment or showed signs of any disease during the week prior to blood collection. Blood samples were collected in EDTA tubes for DNA isolation and CBC analysis (Advia 2120i, Siemens Healthineers, Ballerup, Denmark). A total of 24 CBC traits were measured. The ratios of blood measurements between D25 and D46 were also included as phenotypes. Outliers in the data sets were removed in two steps. Phenotype observations at D25 and D46 that exhibited values deviating from the first or third quartile by more than 1.5 times the interquartile range (IQR) were identified as outliers and excluded from the downstream analysis. After computing trait ratios, a second round of filtering was performed to exclude extreme values caused by denominators approaching zero. Associations between time point and blood parameters were tested using linear mixed‐effects models as implemented in “statsmodel” package (Seabold and Perktold 2010). For each blood parameter, the model included time point, sex, and batch as fixed effects, and animal ID as a random intercept to account for repeated measurements within individuals. Abbreviation, full name for the traits and sample sizes available for analyses after filtering steps are listed in Table S1.

### 2.4 Confounding Factors

One‐way Anova showed that batch and gender affect almost all blood parameters (Table S2); thus, they were used as fixed effect in GWAS. Genotype PCA plots (Figure S2) were generated to check whether inclusion of batch in the model would lead to strong overcorrection for genetic relatedness. It was shown that animals did not tend to cluster by batch (Figure S2), which indicated that batches did not introduce population stratification, that is, batch effects on blood parameters were primarily driven by random environmental variation rather than genetic differences. The two distinct clusters observed in the plot are explained by the admixed structure of the studied population, as pigs inevitably inherit different fractions of their genomes from the Landrace and Yorkshire maternal lines. Heritability estimates and lead SNP effect analyses.

Phenotypic heritability for the 24 blood parameters at two time points, as well as their ratios, was estimated using the genomic‐relationship‐matrix restricted maximum likelihood (GREML) method implemented in the GCTA software (v1.94.1) (Yang et al. 2011). Sex and batch were included as covariates, and original (not imputed) genotype data were used for the analysis.

### 2.5 Genome‐Wide Association Analyses

GWAS was carried out using GCTA and a model for each trait across all SNPs:

$$y=a+\mathrm{bx}+g^{-}+c+e$$

where *y* was a vector of observations on a CBC trait for all individuals, *a* was the mean term, *b* was the additive effect of the SNP to be tested for association, *x* was the SNP genotype indicator variable coded as 0, 1 or 2. The covariance term c represented a fixed batch and gender effect. The “leave‐one‐chromosome‐out” procedure was performed, where *g* ^*−*^ was the accumulated effect of all SNPs based in the genomic relationship matrix (GRM) except those on the chromosome where the candidate SNP was located. The genome‐wide threshold was set to 5 × 10^−8^. Singletons—defined as a lead SNP that lacks a cluster of supporting neighboring SNPs—were removed, as they could represent technical artifacts rather than true biological signals.

### 2.6 Lead SNP Effect Analyses

For the top lead SNPs of each QTL, the phenotypic variance explained was estimated using the formula from (Teslovich et al. 2010):

$$\text{GVar}\left(\%\right)=\frac{2\beta^{2}f\left(1-f\right)\beta^{2}}{2\beta^{2}f\left(1-f\right)+{\left(\mathrm{se}\left(\beta \right)\right)}^{2}2\mathrm{Nf}\left(1-f\right)}*100$$

*β*: effect size of the allele. *f*: frequency of the effect allele. se(*β*): standard error of effect size. *N*: sample size. GVar(%): percentage of phenotypic variance explained by the SNP.

### 2.7 QTL Definition

Independently associated SNPs were identified using the COJO method implemented in GCTA (Yang et al. 2011). The following parameters were applied: a significance threshold of 1.13 × 10^−6^ (‐‐cojo‐p 1.13e‐06), a collinearity threshold of 0.9 (‐‐cojo‐collinear 0.9), a 20 Mb window (‐‐cojo‐wind 20 000), and the stepwise model selection option (‐‐cojo‐slct). Pairwise linkage disequilibrium (LD) (*R* ^2^) was calculated using PLINK (v1.9) (Purcell et al. 2007). Lead SNPs significantly associated with a trait (*p* < 5.0 × 10^−8^) and exhibiting high LD (*R* ^2^ > 0.6) with adjacent genome‐wide significant SNPs were grouped into a single QTL. Since some lead SNPs are associated with multiple traits, we defined a super‐QTL as either a single lead SNP associated with several phenotypes or multiple lead SNPs situated within 1 Mb of one another.

### 2.8 Cross‐Study QTL Comparison

Using Pig QTLdb (Hu et al. 2016) release 53, we assessed whether the QTL regions identified in this study, extended by 500 kb on both sides, overlapped with genomic loci previously associated with the same cell type. Specifically, we first checked whether any previously reported SNPs related to the same hematological trait were located within our QTL intervals. This comparative approach enabled us to identify both SNP‐level and region‐level overlaps, providing evidence for the robustness and potential relevance of our findings and highlighting new findings.

Additionally, the Human GWAS catalog (Cerezo et al. 2025) was used to identify cross‐species QTLs. Genes that were reported in humans with the same hematological cells, that is, erythrocytes, leukocytes, and platelets, were considered as cross‐species candidate genes. Overlap between studies was reported when orthologous genes associated with the same hematological trait were identified both in our study and in the human GWAS catalog. Orthologous genes were identified using g:Profiler (Kolberg et al. 2023).

## 3 Result

### 3.1 Descriptive Statistics of CBC Traits

Descriptive statistics—including mean, standard deviation, minimum, maximum, and distribution—after outlier removal are shown in Figures S3–S5. Almost all blood parameters exhibit significant differences between the two time points. Phenotype correlations are shown in Figure S6. Blood parameters of the same cell type and age are clustered together. Also, mean corpuscular volume (MCV), mean corpuscular hemoglobin content (MCH), as well as all the platelet‐derived phenotypes are clustered together.

### 3.2 Estimates of Heritability

Heritability estimates with standard errors are shown in Table S3. Heritability estimates are moderate to moderately high (0.27–0.62) for erythrocytes, low to moderately low (012–0.43) for leukocytes, except for large unstained cell count (abs_lucs and per_LUC) on D46 for which it is very low (0.03), and moderately low to moderately high (0.37–0.61) for platelets. The heritability of the ratios between D46 and D25 varies across different blood components: It is moderately low (0.25–0.36) for erythrocyte‐related ratios, low to moderately low (0.11–0.29) for leukocyte‐related ratios—except for the large unstained cells ratio, where it is nearly 0—and low to moderately low (0.11–0.47) for platelet ratios.

### 3.3 Genome‐Wide Association Analyses and Estimates of SNP Effects

#### 3.3.1 Erythrocyte Traits

Thirty‐five QTLs were significantly associated with erythrocyte traits at a genome‐wide threshold of 5.0 × 10^−8^ (see Table 1, for position of lead SNP and chromosomal location). The manhattan and quantile‐quantile plots of erythrocytes are shown in Figures S7–S9. These QTLs were grouped into 20 super‐QTLs affecting one or more erythrocyte parameters at one specific time‐point and one super‐QTL, which was associated with four traits across timepoints (super‐QTL 17 on SSC8). Mean corpuscular content (MCH) had the largest number of QTLs, with 12 detected, whereas measured hemoglobin (measHGB) had only 1.

**TABLE 1:** Top SNPs identified by GWAS for significant associations with erythrocyte traits.

QTL ID	Trait	Super‐QTL	Lead SNPs	Freq	Beta	SE	*p*	SNP status	GVar (%)	Bp left	Bp right
1	RBC_D25	1	1:20679855–20 680 354	0.42	0.109	0.01936	1.82e‐08	Lead	1.73	19 148 212	21 661 483
2	MCH_D25	2	1:27416700–27 436 259	0.23	0.0213	0.003382	3.19e‐10	Lead	2.1	25 754 428	29 871 275
3	MCV_D25		1:27416700–27 436 259	0.23	1.02	0.17 973	1.53e‐08	Other	1.69	20 017 740	29 871 275
4	MCV_D25	3	1:51264473–51 336 872	0.36	−0.0236	0.004086	6.54e‐09	Other	1.76	40 869 661	51 351 785
5	MCH_D25		1:51264473–51 336 872	0.36	−1.26	0.217 252	8e‐09	Other	1.79	40 869 661	51 351 785
6	MCH_D25	4	1:113646829–114 121 772	0.14	0.0227	0.003966	1.05e‐08	Other	1.74	106 636 262	125 865 213
7	MCV_D25		1:114485846–114 543 713	0.14	1.27	0.20 899	1.35e‐09	Lead	1.94	110 387 564	119 484 679
8	MCV_D46	5	1:124368304–124 373 114	0.08	1.17	0.209 533	2.62e‐08	Lead	1.89	124 364 338	124 373 114
9	MCHC_D25	6	2:90589993–90 612 529	0.23	−0.132	0.02388	3.02e‐08	Lead	1.64	87 896 797	90 722 574
10	calc_HGB_D46	7	4:9661994–9 671 596	0.06	0.458	0.08388	4.62e‐08	Lead	1.92	9 222 913	9 671 596
11	MCH_D46	8	5:60622418–60 622 720	0.32	−0.0129	0.002283	1.62e‐08	Lead	1.97	59 436 768	60 677 472
12	calc_HGB_D46	9	6:66049349–66 070 676	0.38	−0.208	0.038003	4.1e‐08	Lead	1.94	64 669 958	70 007 810
13	HCT_D46		6:66049349–66 070 676	0.38	−0.0105	0.001839	1.05e‐08	Lead	2.14	64 669 958	70 007 810
14	RBC_D46		6:66049349–66 070 676	0.38	−0.193	0.033939	1.38e‐08	Lead	2.06	64 669 958	70 007 810
15	MCH_D25	10	6:164988749	0.27	0.0208	0.003313	3.45e‐10	Lead	2.09	163 812 258	165 895 067
16	MCV_D25		6:164988749	0.27	0.985	0.175 187	1.9e‐08	Lead	1.67	163 812 258	165 895 067
17	MCH_D46		6:165869214	0.39	0.0128	0.002229	9.51e‐09	Lead	2.03	164 987 340	165 895 378
18	MCH_D46	11	7:23710719	0.06	0.0267	0.004552	4.47e‐09	Lead	2.12	20 740 483	25 368 113
19	HCT_D25	12	7:97163745	0.44	0.00803	0.001313	9.58e‐10	Lead	2.03	97 153 869	97 811 820
20	measHGB_D25		7:97163745	0.44	0.156	0.024512	1.72e‐10	lead	2.21	97 153 869	97 811 820
21	calc_HGB_D25		7:97622770	0.43	0.162	0.025914	4.06e‐10	Lead	2.14	97 153 869	97 811 820
22	MCH_D46	13	8:41511307	0.36	−0.0265	0.003378	5.52e‐15	Lead	3.72	41 436 482	66 963 456
23	RBC_D25	14	8:43218114–43 218 114	0.42	0.108	0.019318	2e‐08	Other	1.72	41 424 861	44 087 637
24	MCH_D25	15	8:49530510–49 556 461	0.17	0.0359	0.004025	4.59e‐19	Lead	4.13	46 164 061	49 611 606
25	MCV_D25		8:49530510–49 556 461	0.16	1.87	0.214 454	3.27e‐18	Lead	3.91	46 164 061	49 611 606
26	MCV_D46		8:49530510–49 556 461	0.17	1.41	0.168 084	5.45e‐17	Lead	4.18	46 164 061	49 611 606
27	MCH_D46	16	8:67660830–67 813 351	0.37	0.0229	0.003301	4.03e‐12	Other	2.94	48 749 181	69 846 812
28	MCH_D25	17	8:74304057–74 311 932	0.14	0.0333	0.004174	1.52e‐15	Other	3.33	70 538 104	75 074 163
29	MCV_D46		8:74304057–74 311 932	0.14	1.22	0.175 382	3.76e‐12	Other	2.91	70 538 104	75 074 163
30	MCV_D25		8:75017268–75 024 421	0.12	1.86	0.234 551	2.14e‐15	Other	3.27	71 330 571	75 113 181
31	RBC_D25		8:75035498–75 035 690	0.13	−0.208	0.032165	1.07e‐10	Lead	2.26	71 366 384	75 113 181
32	MCHC_D46	19	9:24049729–24 070 189	0.44	−0.166	0.024203	6.77e‐12	Lead	2.86	23 581 870	24 298 555
33	MCHC_R		9:24049729–24 070 189	0.44	−0.00905	0.001592	1.32e‐08	Lead	2.17	23 581 870	24 298 555
34	calc_HGB_D25	18	9:137288075	0.19	−0.168	0.0284	3.67e‐09	Lead	1.91	137 287 254	137 297 494
35	MCH_D25	20	12:42631716	0.18	−0.0188	0.00344	4.82e‐08	Lead	1.59	42 231 434	42 897 052

*Note:* Lead SNPs: lead SNP or a range of lead SNPs with the same *p* value are listed. SNP status: The SNPs are either assigned as ‘lead’ SNPs—the most significant variants supported by nearby SNPs—or “other”—i.e., additional independent SNPs on the same chromosome identified by COJO. GVar (%): the proportion of additive genetic variance explained by the top significant SNP. If multiple lead SNPs are identified, the same GVar value applies to each, as they capture the same genetic signal. Bp_left: left border position of the QTL. Bp_right: right border position of the QTL.

#### 3.3.2 Leukocyte Traits

A total of 12 QTLs associated with leukocyte traits were identified at a genome‐wide significance level. Among these, six QTLs were associated with traits measured on D25, while six QTLs were associated with traits measured on D46 (Table 2). The manhattan and quantile‐quantile plots for leukocytes are presented in Figures S10–S12. The 12 QTLs were grouped into nine super‐QTLs. The super‐QTLs 26 and 29 are both related to percentage and absolute basophils and eosinophils.

**TABLE 2:** Top SNPs identified by GWAS for significant associations with leukocyte traits.

QTL ID	Trait	Super‐QTL	Lead SNPs	Freq	Beta	SE	*p*	SNP status	GVar (%)	Bp left	Bp right
36	abs_basos_D46	21	1:260267496	0.33	0.0114	0.001906	2.47e‐09	Lead	2.24	260 189 503	261 110 838
37	per_BASO_D46		1:260267496	0.33	0.0379	0.006947	4.9e‐08	Lead	1.88	260 189 503	261 110 838
38	WBCB_D25	22	3:3433869–3 434 059	0.34	0.539	0.097452	3.28e‐08	Lead	1.65	3 433 823	3 486 306
39	WBCB_D25	23	7:15683616	0.2	−0.791	0.141 657	2.38e‐08	Lead	1.68	15 468 525	15 683 616
40	per_BASO_D25	24	9:47034223–47 038 667	0.089	0.0825	0.015123	4.93e‐08	Lead	1.61	46 734 623	47 317 574
41	per_BASO_D46	25	14:30078190	0.19	0.0481	0.008647	2.57e‐08	Other	1.96	30 010 392	33 120 096
42	per_BASO_D46	26	14:57012970–57 023 681	0.34	0.0437	0.00727	1.88e‐09	Lead	2.28	54 612 164	58 825 122
43	abs_basos_D46	26	14:57859975–57 880 850	0.28	0.0121	0.002187	3.2e‐08	Lead	1.93	56 328 776	58 866 338
44	per_BASO_D46	27	14:107820532	0.46	0.0571	0.009905	8.2e‐09	Other	2.1	106 986 655	108 283 736
45	abs_lymphs_D25	28	16:30271938–30 271 947	0.36	0.488	0.082856	3.9e‐09	Lead	1.88	27 698 660	32 343 334
46	abs_eos_D25	29	17:27527630	0.45	0.0542	0.009631	1.84e‐08	Lead	1.72	26 191 703	27 566 158
47	per_EOS_D25		17:27528146	0.4	0.415	0.073815	1.86e‐08	Lead	1.71	27 188 096	27 566 158

*Note:* Lead SNPs: lead SNP or a range of lead SNPs with the same *p* value are listed. SNP status: The SNPs are either assigned as ‘lead’ SNPs—the most significant variants supported by nearby SNPs—or “other”—i.e., additional independent SNPs on the same chromosome identified by COJO. GVar (%): the proportion of additive genetic variance explained by the top significant SNP. If multiple lead SNPs are identified, the same GVar value applies to each, as they capture the same genetic signal. Bp_left: left border position of the QTL. Bp_right: right border position of the QTL.

#### 3.3.3 Platelet Traits

- Ten QTLs were identified as being associated with platelet traits (Table 3). The manhattan and quantile‐quantile plots for platelets are provided in Figures S13–S15. Of these, six were associated with traits measured on D25, three with traits on D46, and one with the ratio of platelet distribution width (PCDW) between the two time points. The 10 QTLs can be grouped into four super‐QTLs of which one (super‐QTL 32) is related to six platelet traits across the two time points.

**TABLE 3:** Top significant SNPs identified by GWAS for significant associations with platelet traits.

QTL ID	Trait	Super‐QTL	Lead SNPs	Freq	Beta	SE	*p*	SNP status	GVar (%)	Bp left	Bp right
48	PCDW_R	30	5:8618043–8 622 111	0.21	0.043	0.00721	2.44e‐09	Lead	2.4	8 221 259	8 834 727
49	PCT_D25	31	5:19662280	0.15	0.0334	0.005544	1.75e‐09	Other	1.92	19 619 413	22 433 428
50	PLT_D25		5:19662280	0.15	29.3	494 062	2.87e‐09	Other	1.89	19 619 413	22 433 428
51	PCT_D25	32	5:64482450–64 488 583	0.44	−0.0292	0.003658	1.44e‐15	Lead	3.32	64 456 648	64 583 675
52	PCDW_D25		5:64516952	0.42	1.69	0.260 471	9.28e‐11	Lead	2.19	64 456 648	64 583 675
53	PLT_D25		5:64525770	0.44	−26.4	328 232	7.85e‐16	Lead	3.42	64 456 648	64 583 675
54	MPV_D25		5:64525784	0.5	−0.371	0.063139	4.37e‐09	Lead	1.84	64 482 450	65 162 807
55	PCT_D46		5:64525784	0.49	0.0187	0.003392	3.27e‐08	Lead	1.89	64 482 450	65 162 807
56	PLT_D46		5:64525784	0.49	15.4	280 144	3.83e‐08	Lead	1.9	64 482 450	65 162 807
57	MPV_D46	33	7:23071042	0.29	−0.376	0.066784	1.84e‐08	Lead	1.96	23 040 306	26 032 397

*Note:* Lead SNPs: lead SNP or a range of lead SNPs with the same *p* value are listed. SNP status: The SNPs are either assigned as “lead” SNPs—the most significant variants supported by nearby SNPs—or “other”—i.e., additional independent SNPs on the same chromosome identified by COJO. GVar (%): the proportion of additive genetic variance explained by the top significant SNP. If multiple lead SNPs are identified, the same GVar value applies to each, as they capture the same genetic signal. Bp_left: left border position of the QTL. Bp_right: right border position of the QTL.

### 3.4 Cross‐Study Comparison

A comparative analysis of QTL regions from this study and QTL regions reported in previous studies in pigs and humans revealed 15 super‐QTLs overlapping with other pig breeds and 23 super‐QTLs overlapping with human data. Detailed listings of the overlapping QTLs are presented in Tables S4 and S5.

## 4 Discussion

In this study, we provide a genome‐wide overview of the genetic architecture underlying hematological traits in healthy pigs across two developmental stages revealing a polygenic architecture underlying CBC traits with distinct patterns across cell types. The descriptive analyses of CBC traits revealed substantial physiological differences between the two sampling time points, consistent with the major developmental transition occurring around weaning in pigs (George‐Gay and Parker 2003; Bovo et al. 2020). We identified 57 QTLs influencing 17 CBC traits, including loci with time‐independent, time‐specific, and pleiotropic effects. Together, these results highlight both conserved and developmentally dynamic mechanisms underlying hematopoiesis.

Heritability estimates provided insight into the relative contributions of genetic and environmental factors. Erythrocyte traits showed moderate to moderately high heritability, consistent with earlier findings that red blood cell parameters are strongly genetically determined (Bai et al. 2020). Platelet traits also exhibited moderate heritability, indicating a substantial genetic component. In contrast, leukocyte traits displayed lower heritability, particularly for large unstained cells on D46, where heritability was nearly zero. This pattern mirrors previous observations that immune cell traits are highly sensitive to environmental exposures, including microbial challenges and stressors encountered after weaning (Hassan et al. 2022; Zeena Ebraheem mohammed et al. 2020). The consistently higher heritability estimates on D25 compared with D46 support this interpretation: before weaning, animals experience a more uniform environment, allowing genetic effects to explain a larger proportion of phenotypic variance. The lower heritability observed for ratios between time points is likely explained by the removal of the genetically determined pre‐weaning component of the corresponding blood parameters, thereby reducing the remaining genetic variance. At the same time, we observe that heritability estimates for ratios are higher for several white blood cell–related traits (eosinophils, lymphocyte percentage, and neutrophil percentage) compared with D46, which may suggest that the genetic determination of these ratios reflects the robustness of pigs to post‐weaning stressors.

### 4.1 QTLs Overlapping With Previously Discovered Pig QTL

Table S4 summarizes the QTLs identified in this study that overlapped with previously reported loci providing references and breed information for the individual QTL. Super‐QTL 32 has previously been shown to be associated with platelet‐related traits (Bai et al. 2021). Erythrocyte traits showed the largest number of associations, with 35 QTLs grouped into 20 super‐QTLs and one pleiotropic region on SSC8 affecting four traits across both time points (super‐QTL 17). Similar pleiotropic erythrocyte loci have been reported in other pig populations (Jung et al. 2014; Zhang et al. 2013) and in humans (Chen et al. 2020; Kichaev et al. 2019; Vuckovic et al. 2020), suggesting conserved regulatory mechanisms. The particularly high number of QTLs for MCH indicates that this trait captures multiple genetically regulated aspects of erythrocyte physiology, whereas the single QTL detected for measured hemoglobin may reflect a simpler genetic basis or reduced statistical power.

### 4.2 Validation of Genes With Reported Associations to Human Hematological Parameters

Among the candidate genes identified in our GWAS, 147 genes (Table S5) have previously been reported in human studies as significantly associated with the same hematological cell type. This overlap not only validates the reliability of our findings but also suggests the conservation and importance of these genes in regulating hematological parameters. Ten positional/functional candidate genes (*VWF, COQ6, MYB, CD9, KIT, ETV6, MPIG6B, HBS1L, HVCN1*, and *SMIM1*) have previously been reported in humans to be associated with the same cell type. These genes are well‐characterized as regulators of erythropoiesis, thrombopoiesis, or hematopoietic stem cell function (Wang et al. 2018; Fantin 2021; Poggi et al. 2017; Pinho et al. 2018). Five genes (*CXCL9, CXCL10, CXCL11, ADAM10*, and *FLOT1*) have been reported in earlier pig studies, and six genes (*CD63, ZNF410, ENO1, ATF4*, Table 1, and *CACNA1I*) appear to be reported here for the first time in association with pig hematological traits, providing new targets for functional investigation.

### 4.3 Time‐Independent Pleiotropic QTLs

Several SNPs exhibited stable associations with hematological traits across multiple developmental time points. Four time‐independent super‐QTLs (10, 15, 17, and 32) reflect persistent genetic regulation that spans different stages of hematopoiesis and potentially affect multiple blood cell lineages, as shown in Table S6.

Super‐QTL 15 and super‐QTL 17 are associated with erythrocyte traits at both time points reflecting variation in hemoglobin content, cell size, and cell number. The positional candidate genes in super‐QTL 17: *CXCL11*, *CXCL9*, and *CXCL10* are involved in *CXCR3* chemokine receptor binding, a process that plays a crucial role in guiding T cell recruitment to previously inaccessible tissue sites (Karin 2020). Inflammatory and immune signaling pathways influence erythropoiesis and red blood cell function through multiple mechanisms, particularly by regulating erythroid differentiation and anemia under chronic inflammatory conditions (Araki et al. 2020).

Super‐QTL 32 is associated with platelet traits at both time points. The lead SNP is associated with lower PLT and PCT and higher MPV at both time points, suggesting that platelets are being consumed more rapidly, with a compensatory increase in the production of new platelets. A prominent functional candidate gene within this region is von Willebrand factor (*VWF*). VWF plays a key role in hemostasis by forming an adhesive surface at sites of vascular injury, initiating platelet tethering, and subsequently triggering platelet activation and thrombus formation (Chauhan et al. 2008). Interestingly, studies using ex vivo flow models have shown that exposing human megakaryocytes (MKs) to high shear rates can accelerate proplatelet formation and platelet release (Dunois‐Lardé et al. 2009). Moreover, the proportion of circulating platelet precursors in *VWF*−/− mice was 1.8 times higher than in *VWF*+/+ mice, suggesting that VWF also plays a regulatory role in in vivo platelet biogenesis (Poirault‐Chassac et al. 2013). Another candidate gene is *CD9*. Platelet precursor formation is significantly impaired in CD9 knockout mice, and their platelet volume is reduced compared to wild‐type mice (Kono et al. 2008).

### 4.4 Time‐Specific Pleiotropic QTLs

Nine super‐QTLs (super‐QTLs 2, 3, 4, 9, 12, 21, 26, 29, and 31) exhibited time‐specific pleiotropic effects (Table S6). This suggested that these QTLs may either have a transient impact during a specific developmental stage or exert a consistent influence over time through a cumulative mechanism. *HBS1L* and *MYB* are related to MCH_D25 and MCV_D25 (super‐QTL 2). Variants located in the *HBS1L‐MYB* intergenic region on chromosome 6q have been associated with increased fetal hemoglobin levels and changes in red blood cell traits (Stadhouders et al. 2014). These variants can reduce transcription factor binding, affecting long‐range regulation and *MYB* expression (Stadhouders et al. 2014). The *MYB* gene encodes the c‐Myb protein, which plays a role in regulating GATA‐1 activity and proto‐oncogene expression during terminal red cell differentiation (Bartůnek et al. 2003). *ADAM10*, positioned within super‐QTL4, is associated with MCH_D25 and MCV_D25. Conditional deletion of *ADAM10* in endothelial cells results in significantly reduced hemoglobin concentration, hematocrit, and red blood cell counts compared to control mice. Conversely, the relative reticulocyte count is elevated in these mice (Glomski et al. 2011). Super‐QTLs 9 and 12 are associated with HCT, calc_HGB, measHGB, RBC at both D25 and D46. These traits primarily reflect oxygen‐carrying capacity and the process of erythropoiesis. *SMIM1* and *ENO1* are two candidate genes in super‐QTL 9. A common genetic variant of *SMIM1* in humans shows a strong correlation with mean hemoglobin concentration in red blood cells (*p* = 8.6 × 10^−15^) (van der Harst et al. 2012). Supporting its functional significance, targeted suppression of *SMIM1* in zebrafish models results in a modest decrease in red blood cell counts, suggesting that *SMIM1* plays a role in the regulation of red blood cell development (Cvejic et al. 2013). *ENO1* is a key enzyme in the glycolytic pathway. Since red blood cells lack functional mitochondria and rely entirely on glycolysis for ATP production (Martinov et al. 2000), *ENO1* may be essential for their energy metabolism. *COQ6 and ZNF410* are candidate genes in super‐QTL 12. Mutations in the *COQ6* gene can cause primary coenzyme Q10 deficiency‐6 (Wang et al. 2021), which plays a role in protecting red blood cells from oxidative stress (Littarru et al. 1994). *ZNF410* is a remarkably selective transcriptional activator in erythroid cells (Lan et al. 2021) underscoring its potential role in red cell development. *CD63* is a candidate gene in super‐QTL 31 associated with PCT and PLT at D25. Prostate cancer cell‐derived small extracellular vesicles (sEVs) can activate platelets via a CD63‐dependent pathway, leading to platelet aggregation and thrombosis. Activated platelets increase in size, which may contribute to elevated PCT levels. Notably, anti‐CD63 antibodies can inhibit sEV‐induced platelet activation by reducing αIIbβ3 integrin activation and P‐selectin surface expression (Dudiki et al. 2023).

### 4.5 Time‐Specific QTLs

Niteen QTLs are time‐specific QTLs (Table S6). *ETV6* is a candidate gene in QTL11 associated with MCH_D46. *ETV6* has broad functions in early embryonic and adult hematopoiesis, as well as in erythroid and megakaryocytic differentiation and maturation (Feurstein and Godley 2017). QTL 22 and 23 associated with MCH_D46 and RBC_D25 respectively harbor the *KIT* gene. *KIT* is a key regulator in multipotent hematopoietic stem cells, where it governs the proliferation and differentiation of erythroid precursors (Ashman et al. 1991). Mutations in this gene have been implicated in macrocytic anemia across several species, including mice (Russell 1979) and pigs (Johansson et al. 2005). *MPIG6B* and *FLOT1* are candidate genes of QTL 18 and QTL 57, which are associated with MCH_D46 and MPV_D46, respectively. *MPIG6B*, which encodes the immunoreceptor G6b‐B, plays a crucial role in regulating platelet formation, aggregation, and activation. Deficiency or loss‐of‐function mutations in G6b‐B have been linked to thrombocytopenia, anemia, and myelofibrosis in both human and mouse models (Wang et al. 2022). Transcriptomic analysis reveals that *FLOT1* is highly expressed in T cells and platelets and is involved in multiple metabolic pathways (Fu et al. 2025). *HVCN1* is a candidate gene in QTL 41, which is associated with per_BASO_D46. *HVCN1* is highly expressed in basophils and plays a key role in promoting histamine release (Capasso et al. 2011).

### 4.6 Ratio Between D46 and D25

Significant SNPs were identified for the change in PCDW and MCHC between D25 and D46 (Table S6). The QTL associated with PCDW ratio contains *ATF4*, Table 1, and *CACNA1I*, genes linked to the MAPK‐mediated stress responses (KEGG:04010). Their involvement suggests that changes in platelet size distribution may reflect activation of osmotic, oxidative, or calcium‐dependent signaling pathways during the post‐weaning developmental shift (Stadhouders et al. 2014; Bartůnek et al. 2003).

## 5 Conclusion

In conclusion, this study provides a comprehensive genome‐wide overview of the genetic architecture underlying complete blood counts (CBCs) in pigs. We identified 57 QTLs, including time‐specific and time‐independent loci, as well as notable loci associated with D46/D25 ratios. Several QTLs overlapped with those previously reported in different pig breeds, and some were supported by comparable findings in humans, underscoring the conserved nature of hematological regulation. Our results further reveal genetic variation in loci harboring genes known to influence hematological traits in other species; to our knowledge, this is the first evidence for their association with specific blood cell parameters in pigs. These findings not only improve our understanding of the genetic basis of hematological parameters in pigs but also highlight candidate loci with potential relevance to pig health and resilience. Future studies should integrate CBC information with pig production and health traits to explore their practical applications in breeding programs and biomedical research.

## Author Contributions

**Jiahong Sun:** formal analysis, data curation, visualization, writing – original draft, writing – review and editing. **Emil Ibragimov:** formal analysis, methodology, visualization, writing – review and editing. **Malene Kjelin Morsing:** resources, investigation, project administration. **Martin Peter Rydal:** resources, investigation, project administration. **Lise Nikolic Nielsen:** data curation. **Zexi Cai:** data curation, software. Jens Peter Nielsen: resources, investigation, project administration. **Merete Fredholm:** conceptualization, supervision, resources, writing – review and editing, funding acquisition. **Peter Karlskov‐Mortensen:** supervision, methodology, formal analysis, writing – review and editing. All authors reviewed and approved the final manuscript.

## Funding

This work was supported by Novo Nordisk Foundation (NNFSA210073688). China Scholarship Council (CSC202203250011).

## Conflicts of Interest

The authors declare no conflicts of interest.

## Supporting information


**Figure S1:** Distributions of imputation accuracies.
**Figure S2:** Principal component analysis (PCA) plots showing the first two dimensions of the population structure for genotyped animals based on identity‐by‐descent distance.
**Figure S3:** Violin plots for descriptive statistics (mean, standard deviation, maximum, minimum and distribution after removing outliers) for erythrocytes at 25 days and 46 days of age.
**Figure S4:** Violin plots for descriptive statistics (mean, standard deviation, maximum, minimum and distribution after removing outliers) for leukocytes at 25 days and 46 days of age.
**Figure S5:** Violin plots for descriptive statistics (mean, standard deviation, maximum, minimum and distribution after removing outliers) for platelets at 25 days and 46 days of age.
**Figure S6:** Phenotypic correlations between blood parameters at D25 and D46 of age.
**Figure S7:** (A) Manhattan plot of GWAS for erythrocyte traits at D25. The horizontal dashed line indicates the genome‐wide significance threshold (*p* = 5 × 10^−8^). (B) Quantile–quantile (Q–Q) plot showing the observed versus expected −log_10_(*p*) values for the same analysis, illustrating the overall distribution of association signals and potential inflation.
**Figure S8:** (A) Manhattan plot of GWAS for erythrocyte traits at D46. The horizontal dashed line indicates the genome‐wide significance threshold (*p* = 5 × 10^−8^). (B) Quantile–quantile (Q–Q) plot showing the observed versus expected −log_10_(*p*) values for the same analysis, illustrating the overall distribution of association signals and potential inflation.
**Figure S9:** (A) Manhattan plot of GWAS for erythrocyte ratios (D46/D25). The horizontal dashed line indicates the genome‐wide significance threshold (*p* = 5 × 10^−8^). (B) Quantile–quantile (Q–Q) plot showing the observed versus expected −log_10_(*p*) values for the same analysis, illustrating the overall distribution of association signals and potential inflation.
**Figure S10:** (A) Manhattan plot of GWAS for leucocyte traits at D25. The horizontal dashed line indicates the genome‐wide significance threshold (*p* = 5 × 10^−8^). (B) Quantile–quantile (Q–Q) plot showing the observed versus expected −log_10_(*p*) values for the same analysis, illustrating the overall distribution of association signals and potential inflation.
**Figure S11:** (A) Manhattan plot of GWAS for leucocyte traits at D46. The horizontal dashed line indicates the genome‐wide significance threshold (*p* = 5 × 10^−8^). (B) Quantile–quantile (Q–Q) plot showing the observed versus expected −log_10_(*p*) values for the same analysis, illustrating the overall distribution of association signals and potential inflation.
**Figure S12:** (A) Manhattan plot of GWAS for leucocyte ratios (D46/D25). The horizontal dashed line indicates the genome‐wide significance threshold (*p* = 5 × 10^−8^). (B) Quantile–quantile (Q–Q) plot showing the observed versus expected −log_10_(*p*) values for the same analysis, illustrating the overall distribution of association signals and potential inflation.
**Figure S13:** (A) Manhattan plot of GWAS for platelet traits at D25. The horizontal dashed line indicates the genome‐wide significance threshold (*p* = 5 × 10^−8^). (B) Quantile–quantile (Q–Q) plot showing the observed versus expected −log_10_(*p*) values for the same analysis, illustrating the overall distribution of association signals and potential inflation.
**Figure S14:** (A) Manhattan plot of GWAS for platelet traits at D46. The horizontal dashed line indicates the genome‐wide significance threshold (*p* = 5 × 10^−8^). (B) Quantile–quantile (Q–Q) plot showing the observed versus expected −log_10_(*p*) values for the same analysis, illustrating the overall distribution of association signals and potential inflation.
**Figure S15:** (A) Manhattan plot of GWAS for platelet ratios (D46/D25). The horizontal dashed line indicates the genome‐wide significance threshold (*p* = 5 × 10^−8^). (B) Quantile–quantile (Q–Q) plot showing the observed versus expected −log_10_(*p*) values for the same analysis, illustrating the overall distribution of association signals and potential inflation.

**Table S1:** Explanation for the abbreviations used for meassurements in complete blood count.
**Table S2:** One way Anova analysis of batch and gender effects on blood parameters.
**Table S3:** Estimation of heritability for haematological traits.
**Table S4:** Summary of overlapping QTLs between this studis performed by others.
**Table S5:** Overlapping Genes: Associations with Hematological Traits in Pigs and Humans.
**Table S6:** QTL catalog of pig hematological traits.

## Data Availability

The variant data for this study have been deposited in the European Variation Archive (EVA) at EMBL‐EBI under accession number PRJEB102400 (https://www.ebi.ac.uk/eva/?eva‐study=PRJEB102400) (Cezard et al. 2022).
