# Supplementary material for: Genome‐Wide Association Studies for 24 Hematological Traits in Production Pigs Before and After Weaning
Source: Anim Genet. 2026 Mar 26;57(2):e70091. doi: 10.1002/age.70091 (PMC13021266; doi:10.1002/age.70091)
Supplement: Supplementary file 1 — Figure S1: Distributions of imputation accuracies. Figure S2: Principal component analysis (PCA) plots showing the first two dimensions of the population structure for genotyped animals based on identity‐by‐descent distance. Figure S3: Violin plots for descriptive statistics (mean, standard deviation, maximum, minimum and distribution after removing outliers) for erythrocytes at 25 days and 46 days of age. Figure S4: Violin plots for descriptive statistics (mean, standard deviation, maximum, minimum and distribution after removing outliers) for leukocytes at 25 days and 46 days of age. Figure S5: Violin plots for descriptive statistics (mean, standard deviation, maximum, minimum and distribution after removing outliers) for platelets at 25 days and 46 days of age. Figure S6: Phenotypic correlations between blood parameters at D25 and D46 of age. Figure S7: (A) Manhattan plot of GWAS for erythrocyte traits at D25. The horizontal dashed line indicates the genome‐wide significance threshold (p = 5 × 10^−8^). (B) Quantile–quantile (Q–Q) plot showing the observed versus expected −log10(p) values for the same analysis, illustrating the overall distribution of association signals and potential inflation. Figure S8: (A) Manhattan plot of GWAS for erythrocyte traits at D46. The horizontal dashed line indicates the genome‐wide significance threshold (p = 5 × 10^−8^). (B) Quantile–quantile (Q–Q) plot showing the observed versus expected −log10(p) values for the same analysis, illustrating the overall distribution of association signals and potential inflation. Figure S9: (A) Manhattan plot of GWAS for erythrocyte ratios (D46/D25). The horizontal dashed line indicates the genome‐wide significance threshold (p = 5 × 10^−8^). (B) Quantile–quantile (Q–Q) plot showing the observed versus expected −log10(p) values for the same analysis, illustrating the overall distribution of association signals and potential inflation. Figure S10: (A) Manhattan plot of GWAS for leucocyte traits at D25. [file AGE-57-0-s002.docx]

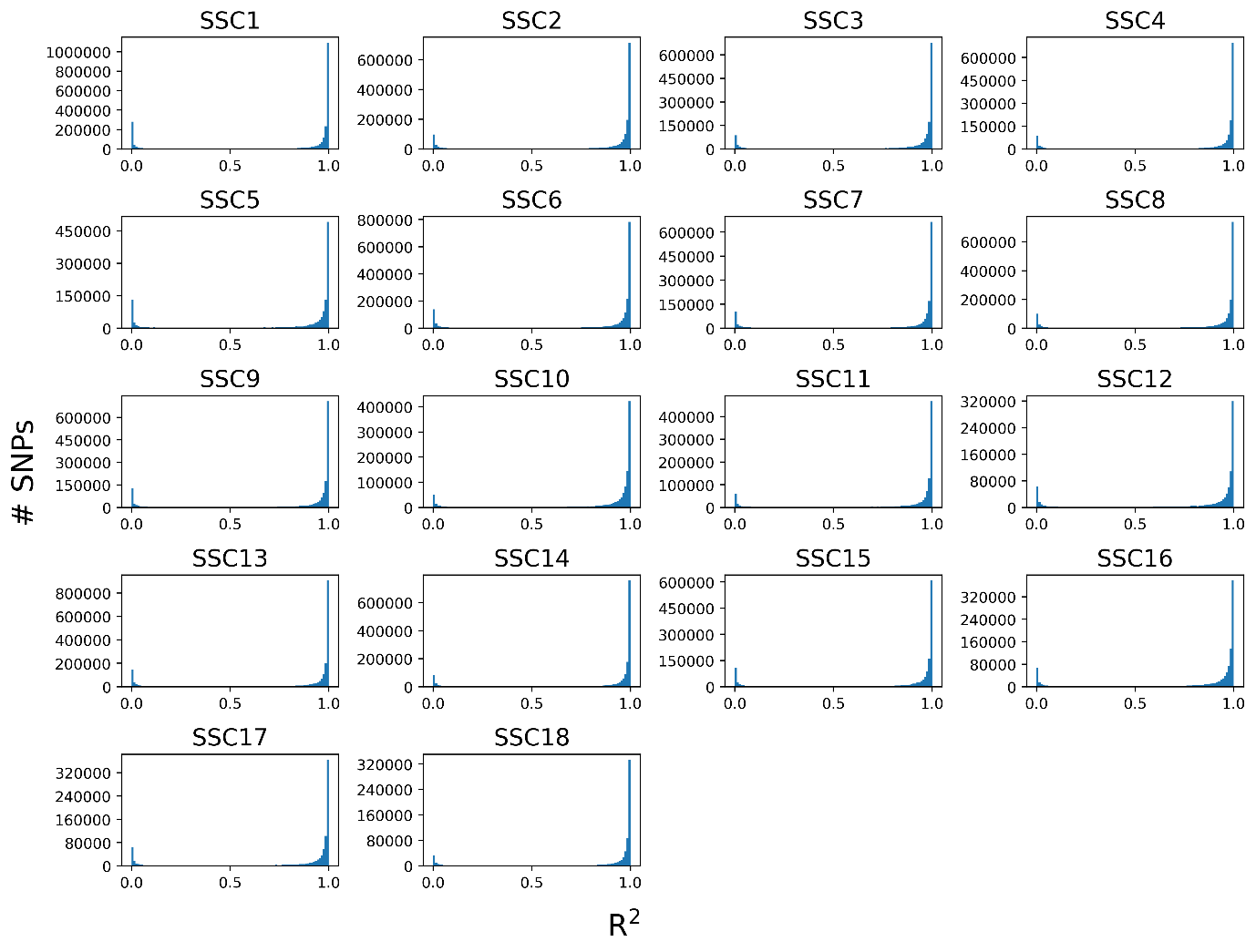


Figure S1 Distributions of imputation accuracies.


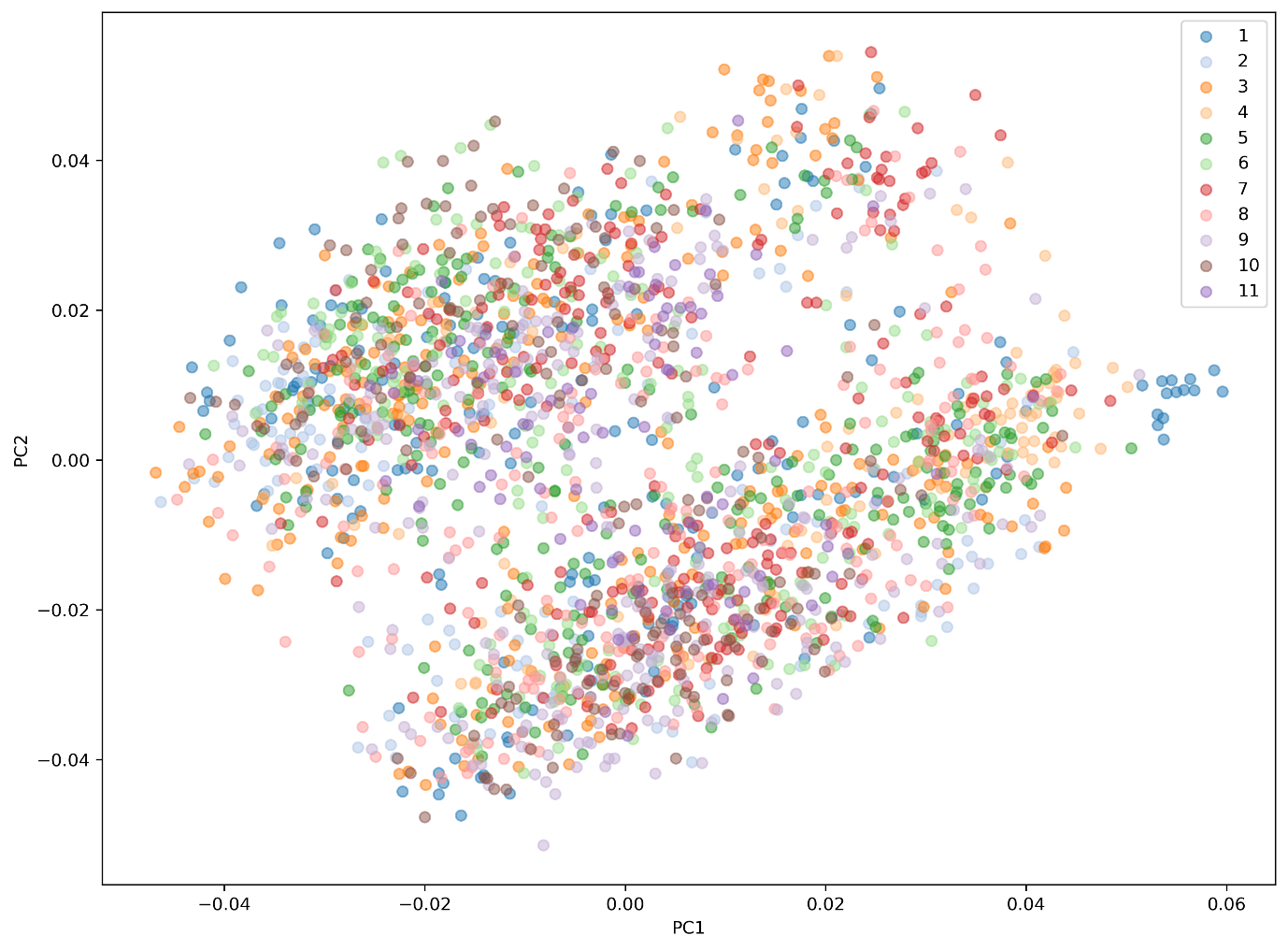


Fig S2 Principal component analysis (PCA) plots showing the first two dimensions of the population structure for genotyped animals based on identity-by-descent distance. Each point represents a genotyped animal, and the colors represent the original batch they belonged to.


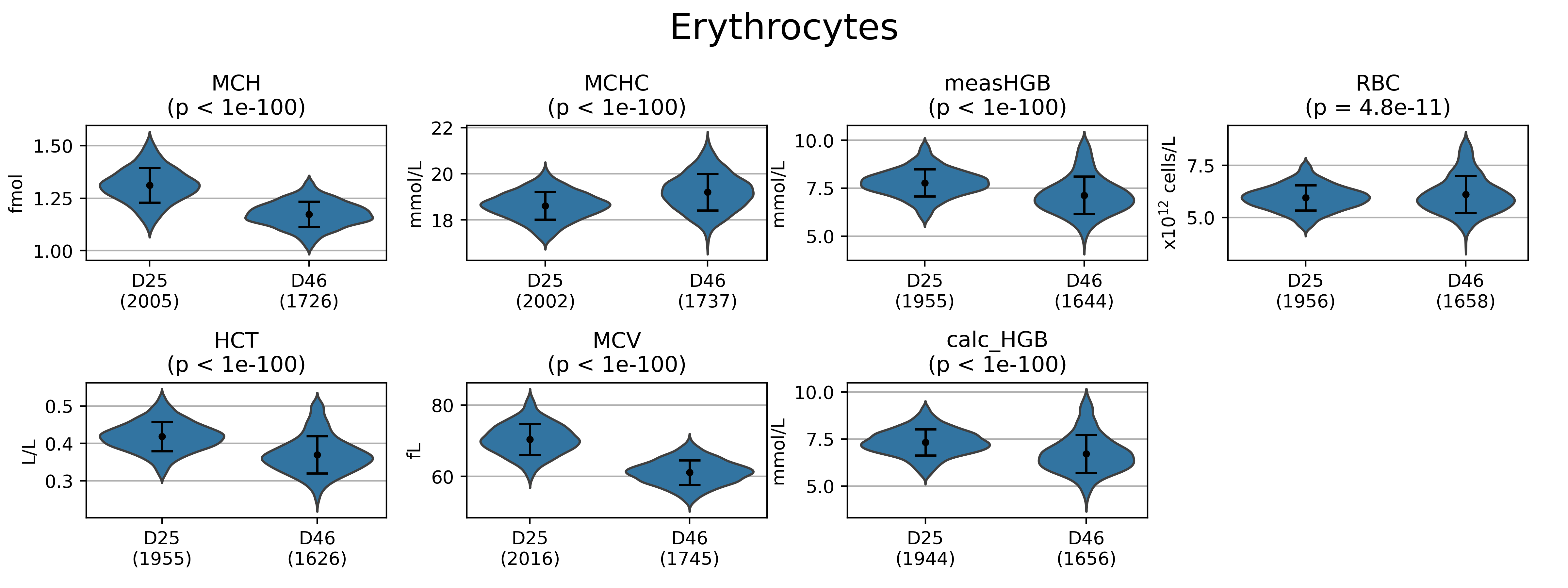


Fig S3 Violin plots for descriptive statistics (mean, standard deviation, maximum, minimum and distribution after removing outliers) for erythrocytes at 25 days and 46 days of age. Numbers in parentheses on the x-axis indicate the number of animals at each time point. P-values of linear mixed model association (as described in “Material and Method”) are shown above the plots. measHGB : measured hemoglobin; calc_HGB: calculated hemoglobin concentration; HCT: hematocrit; MCV: mean corpuscular volume; MCH: mean corpuscular hemoglobin; RBC: red blood cell concentration; MCHC: mean corpuscular hemoglobin concentration.


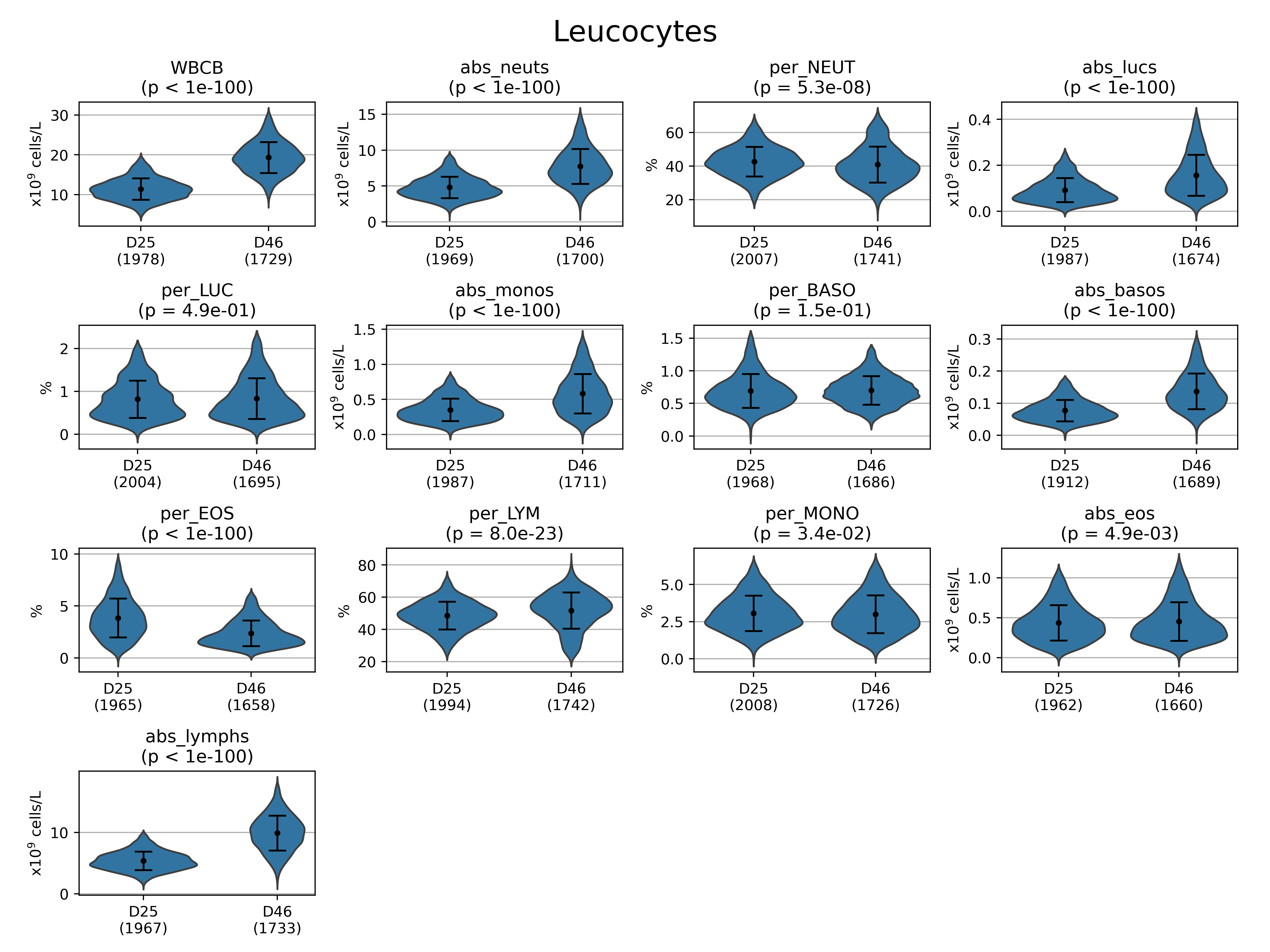


Fig S4 Violin plots for descriptive statistics (mean, standard deviation, maximum, minimum and distribution after removing outliers) for leukocytes at 25 days and 46 days of age. Numbers in parentheses on the x-axis indicate the number of animals at each time point. P values of linear mixed model association (as described in “Material and Method”) are shown above the plots. WBCB: White Blood Cell Count; per_NEUT: Percentage of Neutrophils; per_LYM: Percentage of Lymphocytes; per_MONO: Percentage of Monocytes; per_EOS: Percentage of Eosinophils; per_LUC: Percentage of Large Unstained Cells; per_BASO: Percentage of Basophils; abs_neuts: Absolute Neutrophil Count; abs_lymphs: Absolute Lymphocyte Count; abs_monos: Absolute Monocyte Count; abs_eos: Absolute Eosinophil Count; abs_lucs: Absolute Large Unstained Cell Count; abs_basos: Absolute Basophil Count.


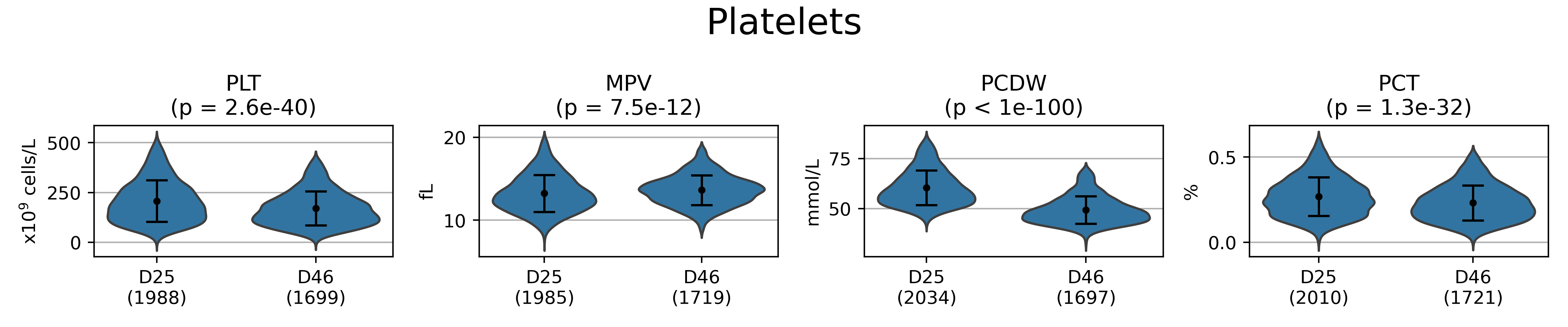


Fig S5 Violin plots for descriptive statistics (mean, standard deviation, maximum, minimum and distribution after removing outliers) for platelets at 25 days and 46 days of age. Numbers in parentheses on the x-axis indicate the number of animals at each time point. P values of linear mixed model association (as described in “Material and Method”) are shown above the plots. PLT: Platelet Count; MPV: Mean Platelet Volume; PCT: Plateletcrit: PCDW: Platelet Distribution Width.


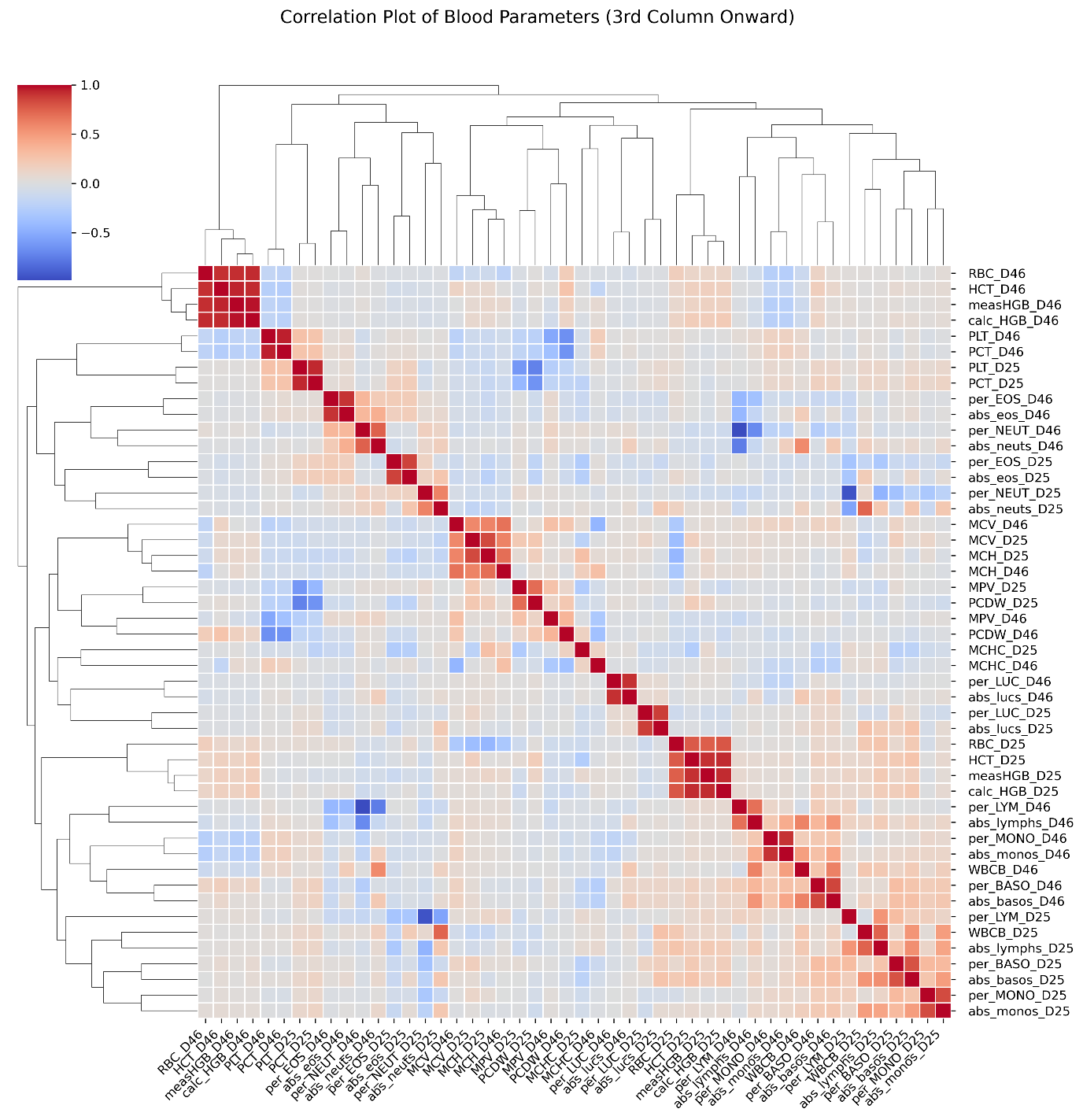


Fig S6 Phenotypic correlations between blood parameters at D25 and D46 of age.


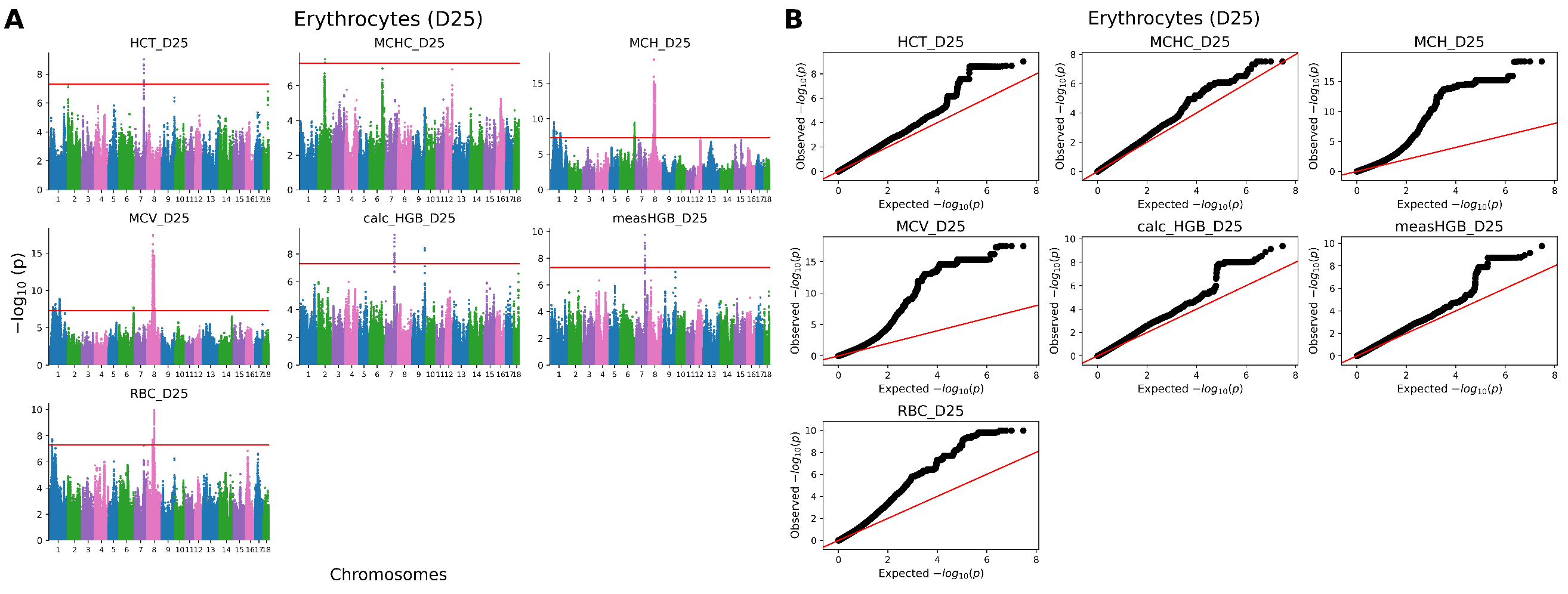


Fig. S7 (A) Manhattan plot of GWAS for erythrocyte traits at D25. The horizontal dashed line indicates the genome-wide significance threshold (P = 5 × 10⁻⁸). (B) Quantile–quantile (Q–Q) plot showing the observed versus expected –log₁₀(P) values for the same analysis, illustrating the overall distribution of association signals and potential inflation.


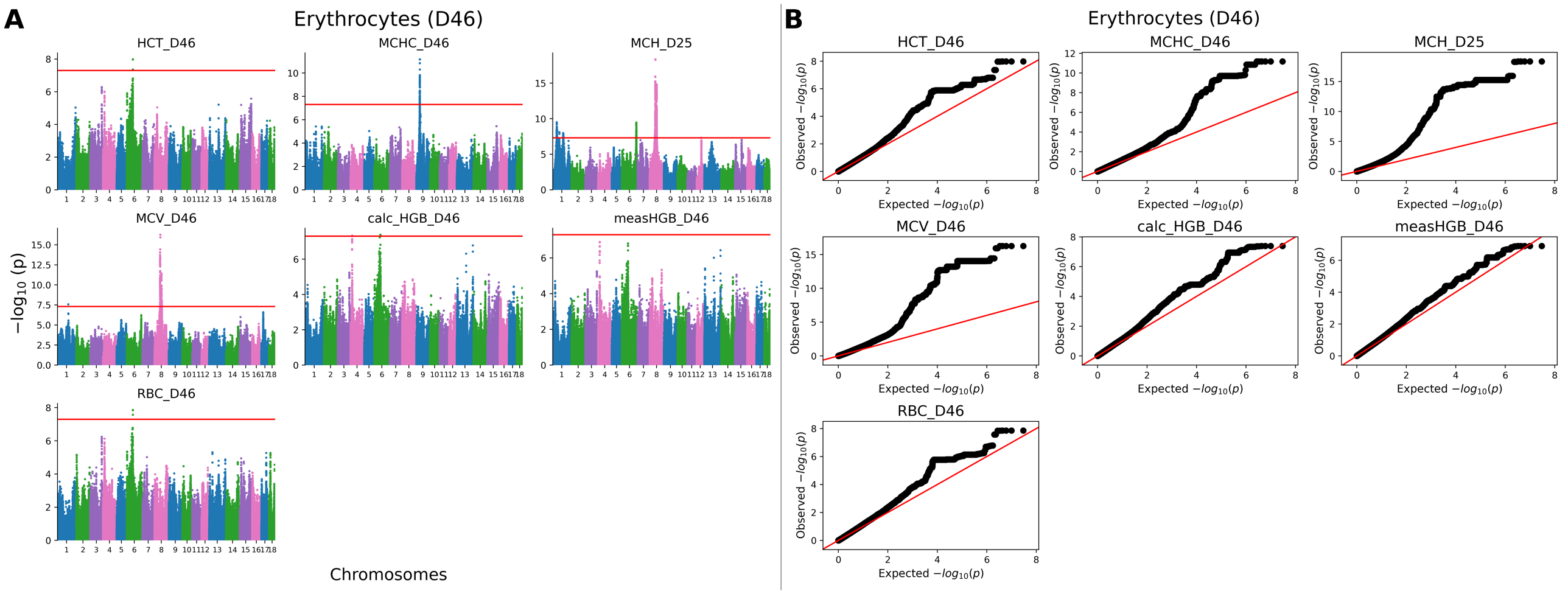


Fig. S8 (A) Manhattan plot of GWAS for erythrocyte traits at D46. The horizontal dashed line indicates the genome-wide significance threshold (P = 5 × 10⁻⁸). (B) Quantile–quantile (Q–Q) plot showing the observed versus expected –log₁₀(P) values for the same analysis, illustrating the overall distribution of association signals and potential inflation.


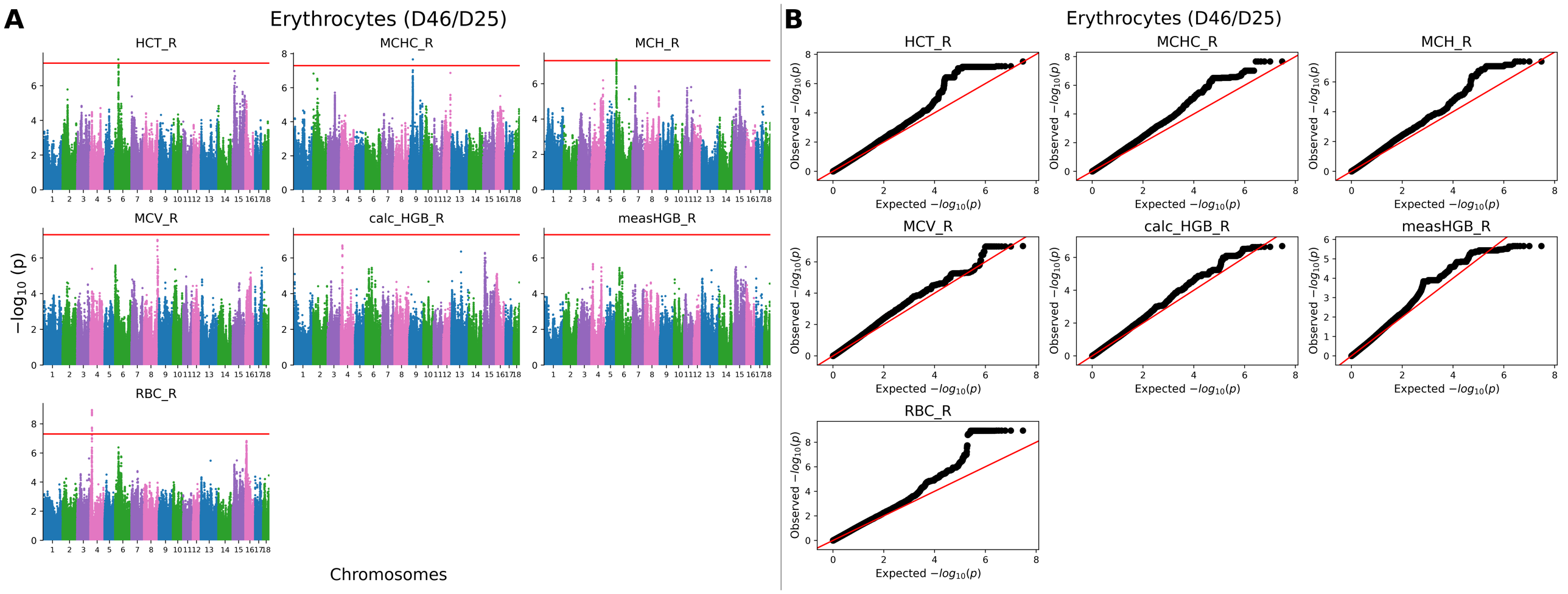


Fig. S9 (A) Manhattan plot of GWAS for erythrocyte ratios (D46/D25). The horizontal dashed line indicates the genome-wide significance threshold (P = 5 × 10⁻⁸). (B) Quantile–quantile (Q–Q) plot showing the observed versus expected –log₁₀(P) values for the same analysis, illustrating the overall distribution of association signals and potential inflation.


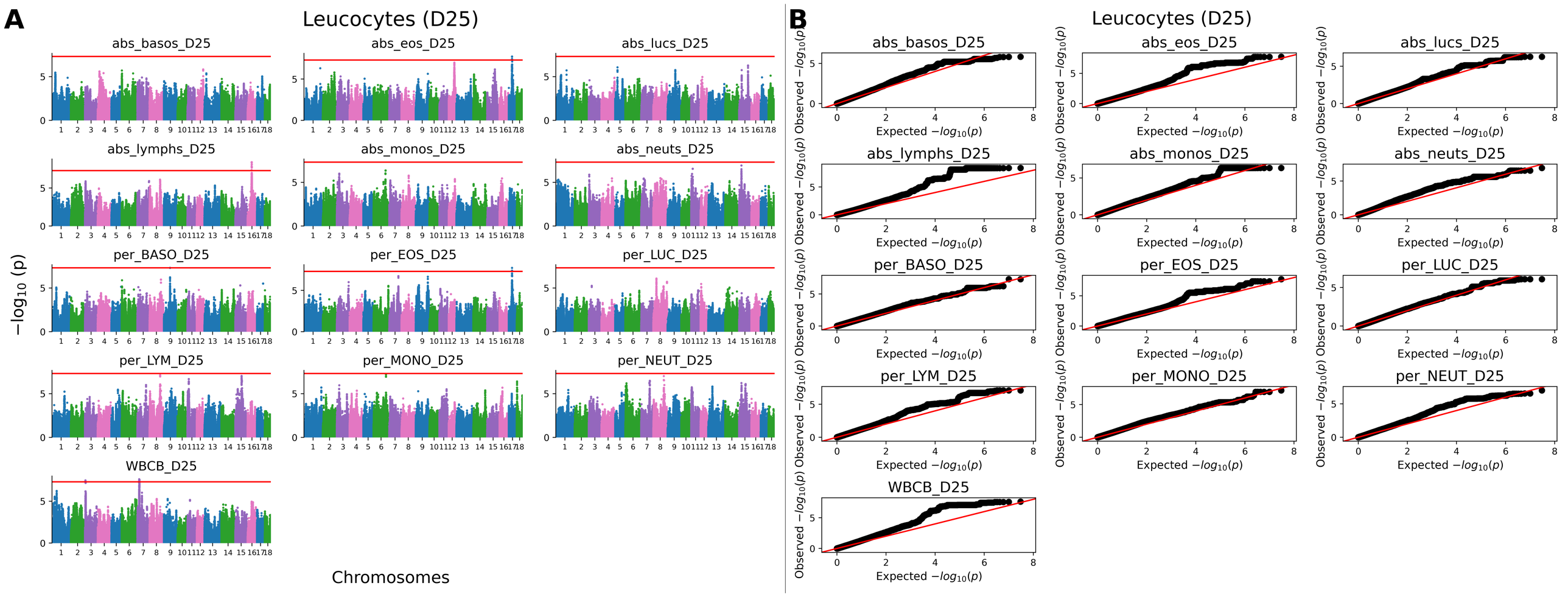


Fig. S10 (A) Manhattan plot of GWAS for leucocyte traits at D25. The horizontal dashed line indicates the genome-wide significance threshold (P = 5 × 10⁻⁸). (B) Quantile–quantile (Q–Q) plot showing the observed versus expected –log₁₀(P) values for the same analysis, illustrating the overall distribution of association signals and potential inflation.
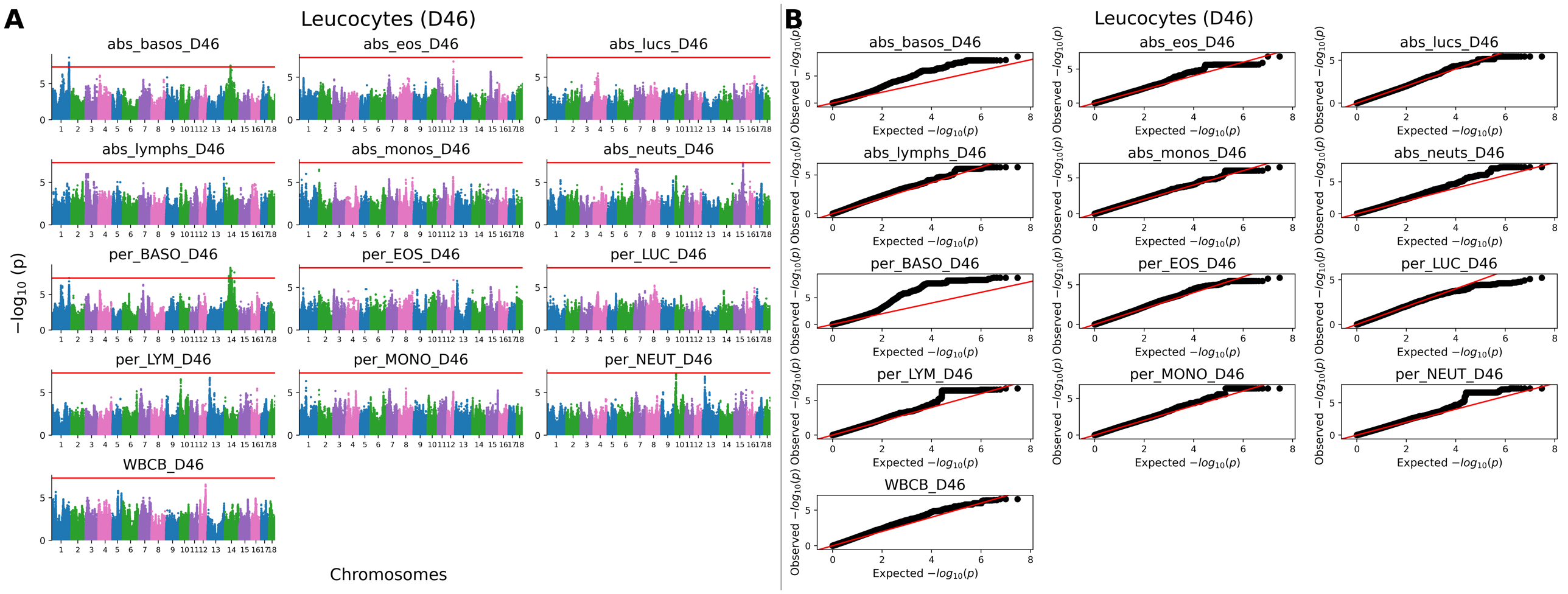


Fig. S11 *(A)* Manhattan plot of GWAS for leucocyte traits at D46. The horizontal dashed line indicates the genome-wide significance threshold (P = 5 × 10⁻⁸). *(B)* Quantile–quantile (Q–Q) plot showing the observed versus expected –log₁₀(P) values for the same analysis, illustrating the overall distribution of association signals and potential inflation.
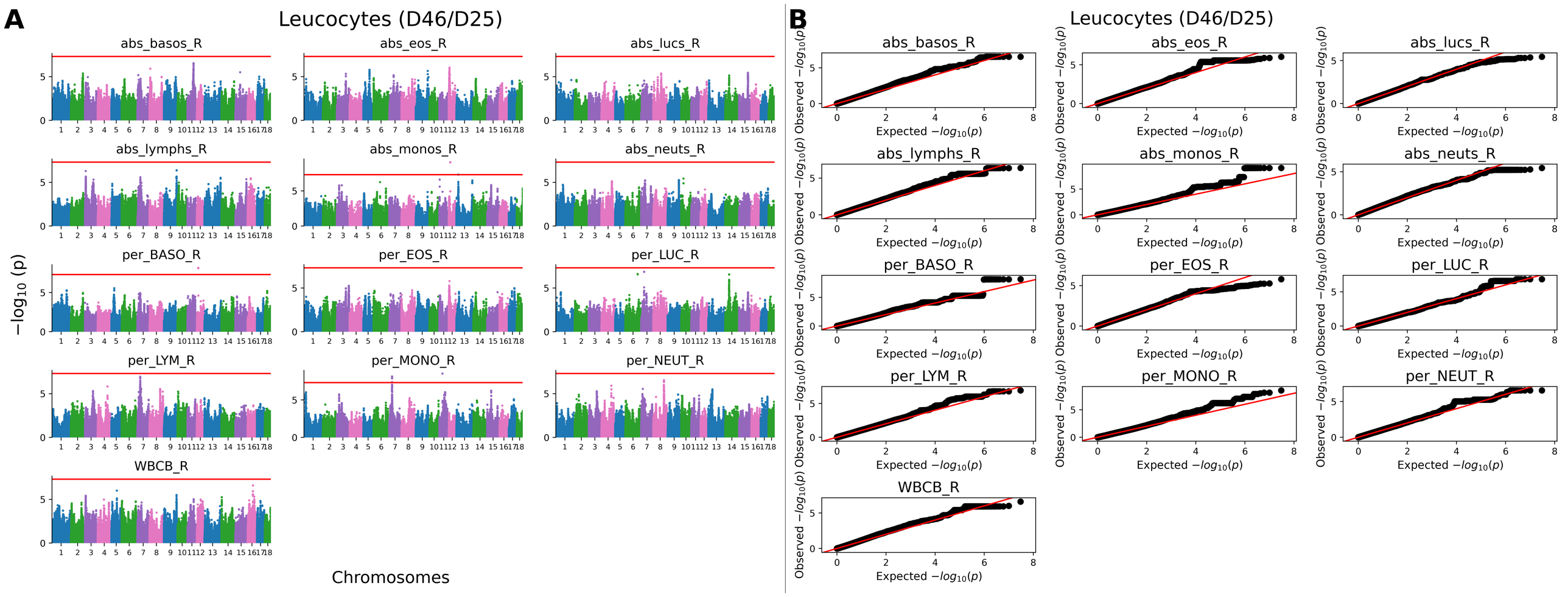


Fig. S12 (A) Manhattan plot of GWAS for leucocyte ratios (D46/D25). The horizontal dashed line indicates the genome-wide significance threshold (P = 5 × 10⁻⁸). (B) Quantile–quantile (Q–Q) plot showing the observed versus expected –log₁₀(P) values for the same analysis, illustrating the overall distribution of association signals and potential inflation.


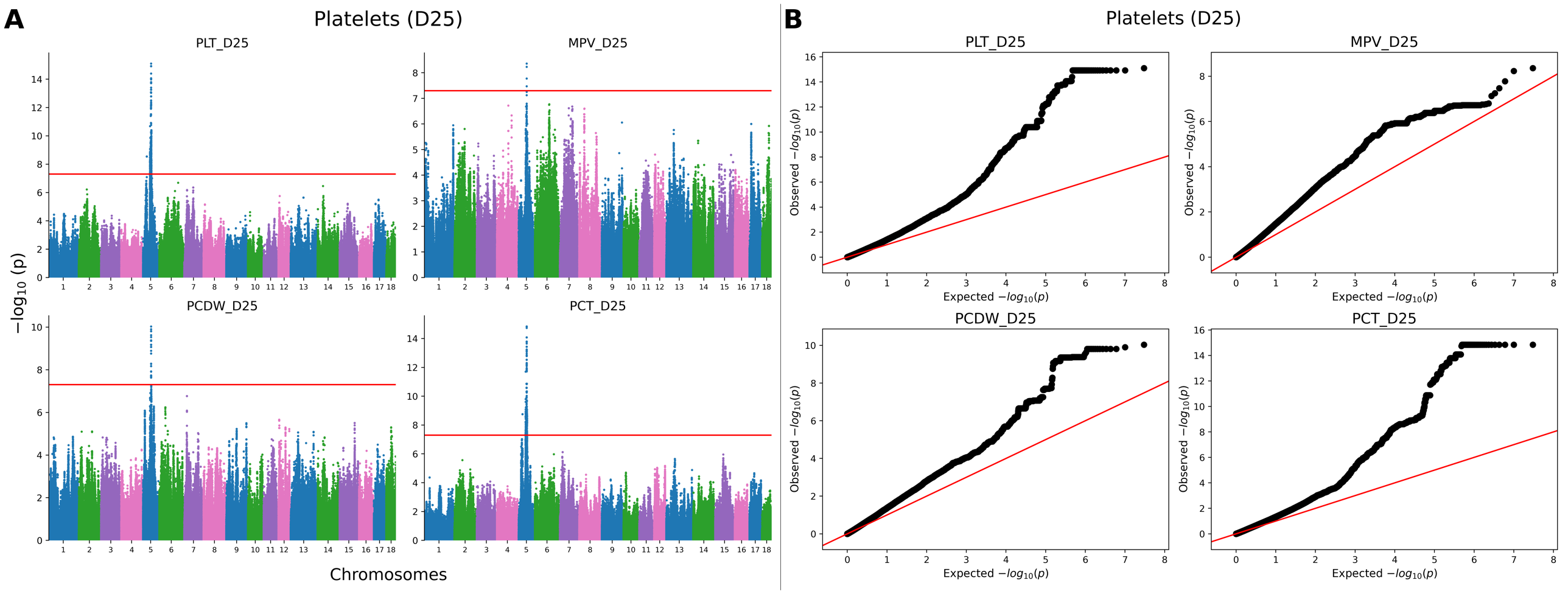


Fig. S13 (A) Manhattan plot of GWAS for platelet traits at D25. The horizontal dashed line indicates the genome-wide significance threshold (P = 5 × 10⁻⁸). (B) Quantile–quantile (Q–Q) plot showing the observed versus expected –log₁₀(P) values for the same analysis, illustrating the overall distribution of association signals and potential inflation.


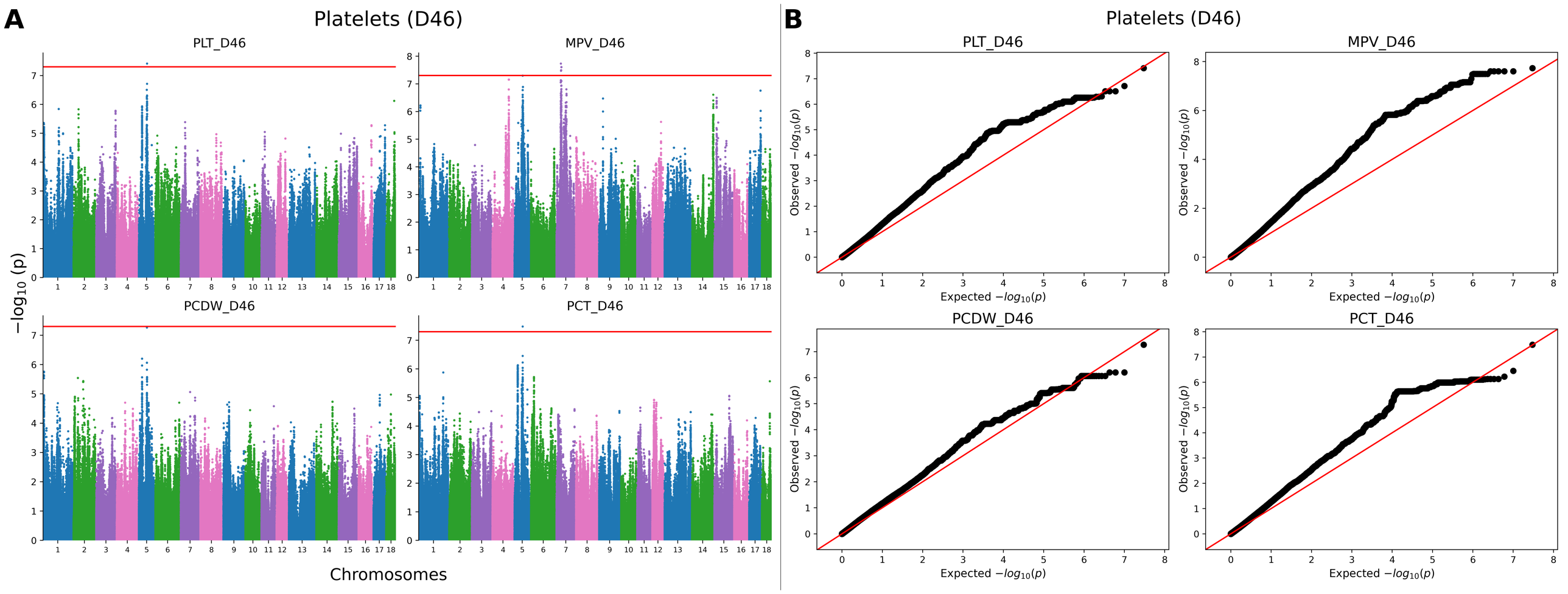


Fig. S14 (A) Manhattan plot of GWAS for platelet traits at D46. The horizontal dashed line indicates the genome-wide significance threshold (P = 5 × 10⁻⁸). (B) Quantile–quantile (Q–Q) plot showing the observed versus expected –log₁₀(P) values for the same analysis, illustrating the overall distribution of association signals and potential inflation.


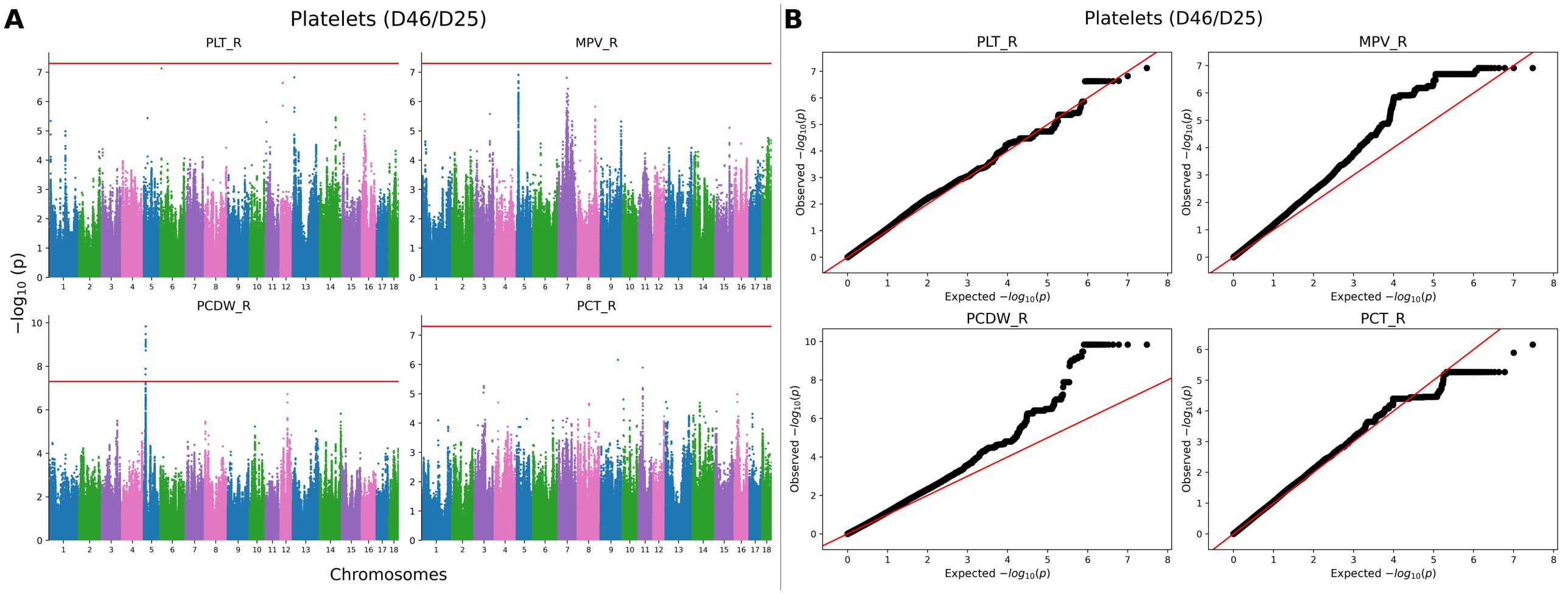


Fig. S15 (A) Manhattan plot of GWAS for platelet ratios (D46/D25). The horizontal dashed line indicates the genome-wide significance threshold (P = 5 × 10⁻⁸). (B) Quantile–quantile (Q–Q) plot showing the observed versus expected –log₁₀(P) values for the same analysis, illustrating the overall distribution of association signals and potential inflation.
